# Utility of intracellular enhancement for improving hepatic lesion visibility at gadoxetic acid-enhanced hepatobiliary-phase MRI

**DOI:** 10.1016/j.ejro.2026.100814

**Published:** 2026-08-19

**Authors:** Shogo Maeda, Yuko Nakamura, Toru Higaki, Takashi Nishihara, Masahiro Takizawa, Yoshitaka Bito, Hirokazu Asaka, Kenji Kodakari, Dara Fonseca, Shota Kondo, Yukiko Honda, Yuji Akiyama, Tomokazu Kawaoka, Masataka Tsuge, Shiro Oka, Kazuo Awai

**Affiliations:** a Diagnostic Radiology, Hiroshima University, Japan; b FUJIFILM Corporation, Japan; c Department of Diagnostic Imaging, Hokkaido University Graduate School of Medicine, Japan; d Department of Radiology, Hiroshima University Hospital, Japan; e Department of Gastroenterology, Hiroshima University, Japan; f Hiroshima Prefectural Hospital Organization, Japan

**Keywords:** Magnetic resonance imaging, Gd-EOB-DTPA, Liver neoplasms, Image enhancement, Contrast media

## Abstract

**Purpose:**

To investigate the utility of the intracellular enhancement (ICE) technique that suppresses extracellular signal component for shortening the gadoxetic acid (EOB)-enhanced hepatobiliary-phase (HBP) delay time while maintaining lesion visibility.

**Methods:**

In this prospective study, patients who underwent EOB-enhanced MRI were screened and those with HBP hypointense hepatic lesions were included. HBP images were acquired at 10 and 20 min post-EOB injection, with and without ICE (i^+^- and i^-^-10 and 20 min HBP). One representative lesion per patient was evaluated. The lesion-to-liver contrast ratio (CR) was calculated. Two radiologists assessed overall image quality and lesion visibility on a five-point scale. The primary outcome was lesion visibility, which was assessed qualitatively using subjective lesion visibility scores and quantitatively using CR. Differences between paired groups were determined by using a two-sided Wilcoxon signed-rank test.

**Results:**

Eighty-one patients were included. Overall image quality was significantly worse with ICE at both 10- and 20-min HBP (10 min: mean 3.8 vs 3.1; 20 min: 4.0 vs 3.3; both p < 0.01). There was no significant difference in the CR between i^+^-10 min and i^-^-20 min HBP (median 1.58 vs 1.54; p = 0.48). Furthermore, lesion visibility scores on i^+^-10 min HBP were significantly higher than those on i^-^-20 min HBP (mean 4.1 vs 3.9; p < 0.01).

**Conclusion:**

ICE has the potential to reduce delay time without compromising lesion visualization; however, further studies with larger sample size.

## Highlights

• Reducing HBP delay while maintaining lesion visibility remains challenging.
• ICE suppresses signals from the extracellular space.
• ICE improves the visibility of hypointense hepatic lesions on the HBP.
• ICE may enable shorter HBP delays while maintaining lesion visibility.

## 1 Introduction

Due to its hepatocyte-specific uptake, gadoxetic acid (Gd-EOB-DTPA: EOB)-enhanced hepatobiliary-phase (HBP) MRI is highly sensitive for detecting hepatic lesions [1]. Therefore, it has been strongly recommended for detecting malignant tumors such as hepatocellular carcinoma (HCC) and liver metastases [2], [3], [4], [5]. However, the diagnostic performance of EOB–enhanced MRI in the HBP depends on hepatocyte function. In patients with severe liver dysfunction or biliary disease, reduced EOB uptake can lead to globally diminished HBP enhancement and decreased lesion-to-liver contrast [5]. In addition, the conventional 20-min HBP delay required for adequate contrast limits clinical throughput, as only 61% of patients have been reported to maintain sufficient liver enhancement on 10-min HBP images compared with conventional 20-min HBP images [6]. Therefore, there is a clinical need for techniques that maintain lesion conspicuity while enabling shorter HBP delay times without compromising diagnostic performance.

Given these limitations, techniques that improve lesion-to-liver contrast, thereby improving lesion visibility on HBP and allowing for shorter HBP delay times without compromising diagnostic performance, are increasingly desired. Several strategies have been proposed to improve lesion conspicuity on HBP images, including increasing the HBP flip angle, which enhances lesion-to-liver contrast [7]. During the HBP, liver enhancement reflects both intracellular uptake of EOB by functioning hepatocytes and residual extracellular contrast material. In normal liver parenchyma, both components contribute to signal intensity. In contrast, because most hepatic lesions lack functioning hepatocytes, their enhancement is derived predominantly from the extracellular component. Consequently, suppression of the extracellular signal component selectively increases the relative contribution of hepatocyte-specific enhancement of the liver parenchyma, thereby increasing lesion-to-liver contrast and improving lesion conspicuity. However, the currently available approaches increase overall liver enhancement rather than selectively enhancing the hepatocyte-specific component of contrast enhancement. Therefore, there is a need for strategies that selectively enhance the hepatocyte-specific component while minimizing the extracellular component.

Based on this concept, the intracellular enhancement (ICE) technique was recently developed. ICE uses a motion-sensitized driven equilibrium pulse to suppress the extracellular space component [8]. Previous studies demonstrated that ICE improves the hepatocyte-specific component of hepatobiliary enhancement and is useful for liver function assessment [9]. However, its effect on hepatic lesion conspicuity has not been investigated. Furthermore, whether ICE can compensate for reduced hepatobiliary enhancement at earlier imaging time points, thereby enabling shortening of the HBP delay, remains unknown. Based on its imaging mechanism, we hypothesized that suppression of the extracellular signal component would increase lesion-to-liver contrast, thereby improving lesion conspicuity on HBP images while permitting shorter HBP delay times. Therefore, the aim of this study was to determine whether 10-min ICE HBP imaging could maintain hepatic lesion visibility compared with conventional 20-min HBP imaging. Hepatic lesion visibility was assessed qualitatively using lesion visibility scores and quantitatively using lesion-to-liver contrast ratios.

## 2 Materials and methods

### 2.1 Patients

This prospective study was approved by the Human Ethics Review Committee of our institute (E2020–2169); prior signed informed consent was obtained from all study subjects. Patient records and information were anonymized and de-identified prior to analysis.

Consecutive patients who provided written informed consent and underwent EOB-enhanced MRI at our institution between November 2022 and November 2024 were enrolled. Patients with hepatic lesions showing hypointensity on HBP images were included in the final analysis. Exclusion criteria were pediatric patients, because the HBP imaging protocol differs from that used in adults (e.g., respiratory-gated acquisition); patients who failed to fulfill all HBP scanning protocols; and contraindications for MRI (e.g., cardiac pacemakers or metal implants). Final diagnoses were established on the basis of histopathological findings or imaging follow-up, as appropriate. Because the primary aim was to evaluate lesion visibility rather than lesion characterization, lesions with different etiologies were pooled when they showed hypointensity on HBP images.

Hypervascular HCCs were diagnosed conclusively upon pathological proof of the tumor burden obtained after partial hepatectomy or the imaging-based practical guidelines proposed by the American Association for the Study of Liver Diseases [10], i.e., wash-in in the arterial phase and washout in the delayed phase of dynamic CT. HBP hypointense nodules without arterial-phase hyperenhancement (APHE) are defined as those appearing hypovascular in the arterial phase and hypointense in the HBP on EOB-enhanced MRI scans [11]. These nodules are frequently observed in patients with chronic liver disease and are categorized as borderline nodules such as dysplastic nodules and early HCCs which serve as indicators of hypervascular HCC [12]. Metastatic tumors were diagnosed on imaging either by an increase in size or disappearance after chemotherapy on follow-up studies in patients with a recent history of malignancy, or by the presence of multiple lesions with a clearly identified malignant primary tumor and elevated tumor markers. Hemangiomas were diagnosed using the following imaging features: obviously high signal intensity (SI) on T2-weighted images and the typical enhancement pattern on dynamic CT or MRI scans.

Two board-certified radiologists (SM and YN with 6 and 21 years of experience, respectively) confirmed the presence and location of hepatic lesions on contrast-enhanced CT images, EOB-enhanced MR images, and PET images obtained by using fluorine 18 fluorodeoxyglucose. Pathologic findings obtained at definitive surgery were also considered.

### 2.2 MRI protocol

#### 2.2.1 Intracellular enhancement technique

The motion-sensitized driven-equilibrium (MSDE) pulse suppresses signals from the blood flow by adding motion-proving gradients (MPGs) with a low b-value [13], [14]. This increases hepatocyte-specific enhancement during HBP by suppressing signals from the blood flow in the extracellular space. The ICE technique combines MSDE and the fat-saturated T1-weighted gradient-echo nature of the sequence (TIGRE) to increase hepatocyte-specific contrast enhancement on HBP images [8]. Briefly, the MSDE pulse is composed of 3 composite radiofrequency hard pulses, MPGs, and spoiler gradients. The fat saturation pulse is comprised of chemical shift-selective pulses and spoiler gradients. The MSDE pulse (b-value 60 s/mm^2^, duration 21 ms) is applied immediately before the fat saturation pulse (duration 100 ms). The 3D RF-spoiled gradient-echo sequence is applied after the MSDE pulse and the fat saturation pulses to acquire T1-weighted signals modified by the preparation pulses.

#### 2.2.2 Image acquisition

Scanning was on a 3 T MRI instrument (FUJIFILM Corporation, Tokyo, Japan) using a 28-channel coil. Twenty-five μmol/kg of EOB (EOB-Primovist, Bayer Yakuhin, Osaka, Japan) was injected intravenously at a rate of 2.0 mL/s followed by 20 mL of saline delivered at the same rate using a power injector (Sonic Shot 50; Nemoto-Kyorindo, Tokyo, Japan). HBP imaging was performed 10 and 20 min after the contrast injection, with and without the ICE technique (i^+^- and i^-^−10 and 20 min HBP). Scanning was with TIGRE with parallel imaging (rapid acquisition through a parallel imaging design; RAPID, FUJIFILM Corporation). The acquisition time remained almost the same when ICE was used.

Diffusion-weighted images of the liver feature low resolution, noise, and artifacts [15]. As the MSDE pulse used in the ICE technique suppresses signals from the blood flow by adding MPGs with a low b-value [13], [14], ICE may degrade the image quality due to the MSDE pulse. Wavelet denoising with geometry factor weighting (g-denoising) can reduce the image noise by adapting to spatially varying noise levels induced by parallel imaging; it yields a better image quality than conventional HBP imaging [16]. Thus, g-denoising was added to our protocols for HBP scanning with and without ICE.

The other scan parameters for HBP were section thickness and interval 4.0 mm, TR/TE 4.0 msec/1.8 msec, flip angle 15°, field of view 36 cm, matrix 320 × 224, and parallel imaging factor 2.2 (2.0 for phase direction and 1.1 for slice direction). Parallel imaging reconstruction was performed in the image space domain because g-denoising can be applied to parallel imaging reconstruction performed in the image- but not in the k-space domain [16]. Although dynamic EOB-enhanced MRI scans were obtained in the clinical studies they were not evaluated.

### 2.3 Image analysis

#### 2.3.1 Qualitative image analysis

Images were presented in random order and independently evaluated by two radiologists (SK and YH with 7 and 23 years of experience, respectively) who were blinded to the acquisition protocol, acquisition time point, and clinical information. When discrepant scores occurred, consensus was reached through discussion, and the consensus scores were used for the final statistical analyses. Both readers received standardized instructions and were trained using image sets from five patients who were not included in the study. They ranked artifacts and overall image quality based on a previously reported grading system (Likert Scale). For artifacts: 1 = extensive artifacts, non-diagnostic; 2 = severe artifacts, image degraded but interpretable; 3 = moderate artifacts, some effect on diagnostic quality; 4 = minimal artifacts, no effect on diagnostic quality; 5 = no artifact. For overall image quality: 1 = unacceptable, 2 = suboptimal, 3 = acceptable, 4 = good, 5 = excellent [17]. Both radiologists also recorded the visibility of hepatic lesions using a five-point Likert scale where 1 is definite artifact mimicking a lesion; 2 is probable artifact mimicking a lesion; 3 is subtle lesion; 4 is well-visualized lesion with poorly visualized margins; and 5 is well-visualized lesion with visualized margins. As it is more difficult to detect small tumors than large tumors, the visualization of small tumors is of clinical importance and requires improvement. In patients with multiple hepatic lesions, we only evaluated the smallest lesion [18].

#### 2.3.2 Quantitative image analysis

A board-certified radiologist (SM with 6 years of experience) performed quantitative analysis. For SI measurements a region of interest (ROI) was placed on the hepatic lesions and the surrounding hepatic parenchyma. The ROIs for hepatic lesions encompassed the whole lesion and were placed only in lesions that were included in the qualitative image assessment. The SI of the surrounding hepatic parenchyma was recorded as the mean value of 4 ROIs in the right anterior- and posterior-, and the left medial, and lateral segments of the liver. Areas of focal changes in hepatic parenchyma, large vessels, and prominent artifacts were carefully avoided. The same radiologist calculated the contrast ratio (CR) as CR = ROI_LIV_/ROI_LES,_ where ROI_LIV_ and ROI_LES_ represent the mean SI of the hepatic parenchyma and lesion, respectively.

### 2.4 Statistical analysis

All statistical analyses were performed using JMP Student Edition version 18 (SAS Institute, Cary, NC). To evaluate the effect of ICE on lesion visibility, CR values and subjective lesion visibility scores were compared between i^-^-HBP and i^+^-HBP at both 10 min- and 20 min. Subsequently, CR values and subjective lesion visibility scores obtained from i^-^ and i^+^−10 min HBP images were compared with those from the conventional i^-^−20 min HBP images to assess whether ICE enables a reduction in delay time without compromising lesion visibility.

Differences between paired groups were analyzed using the two-sided Wilcoxon signed-rank test. For the subsequent analyses comparing 10-min HBP images with the conventional i^-^−20 min HBP images, p-values were adjusted for multiple comparisons using the Bonferroni method, and a p-value < 0.025 was considered statistically significant. Otherwise, a p-value < 0.05 was considered statistically significant. Subgroup analyses were also performed according to the patients’ Child–Pugh classification.

We calculated interobserver agreement using the weighted k statistic to evaluate agreement between the two readers and interpreted where excellent: > 0.90, good: 0.75 to < 0.90, moderate: > 0.50 to < 0.75, and poor: < 0.50 [19].

## 3 Results

### 3.1 Patient population

**Fig. 1:**
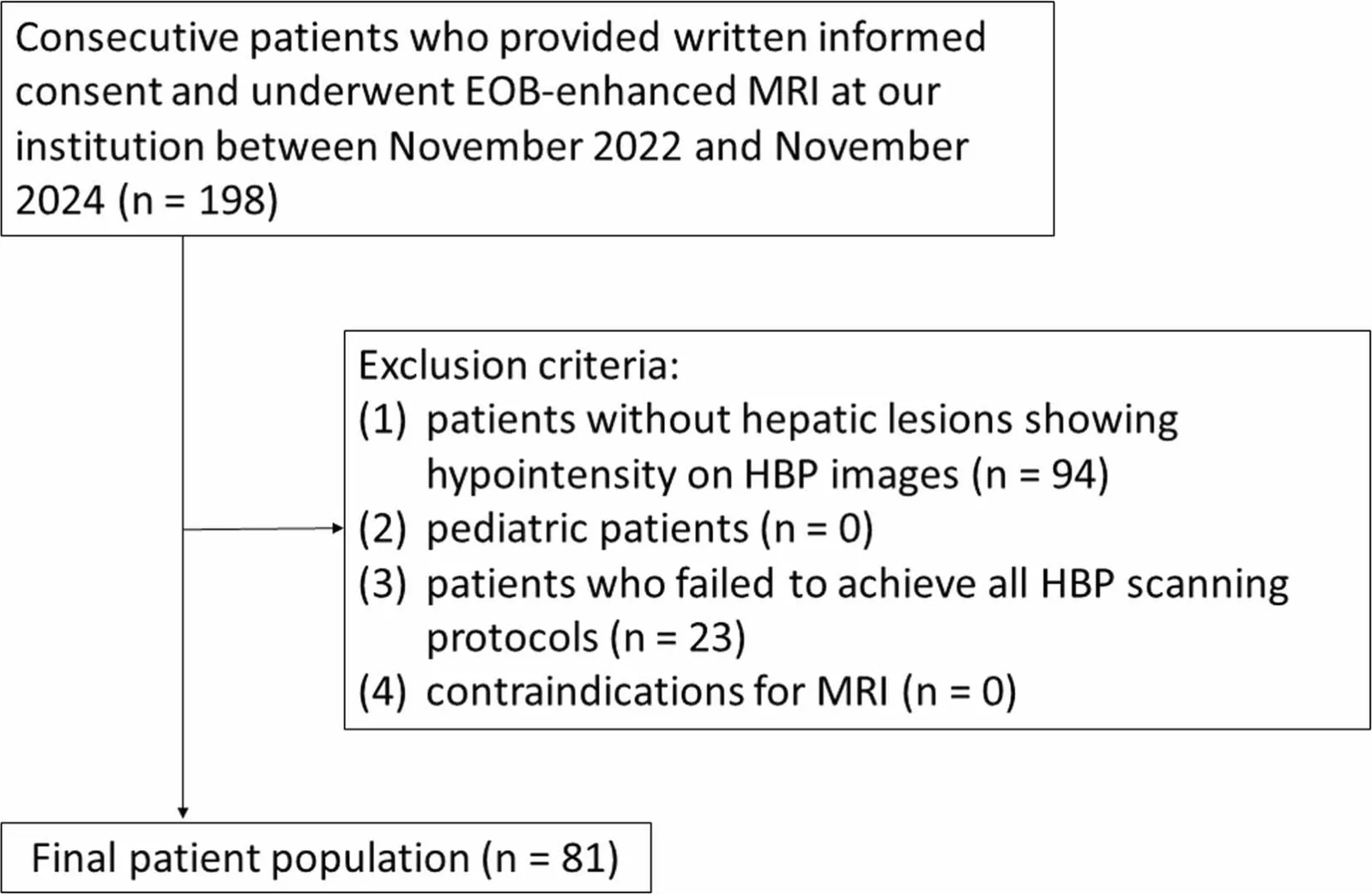
Flowchart of case enrollment.

A total of 198 consecutive patients were prospectively enrolled during the study period. Of these, 94 patients were excluded because they had no hepatic lesions showing hypointensity on HBP images. An additional 23 were excluded because one or more required HBP image sets had not been transferred to the image server owing to technical workflow errors. Consequently, 81 patients (59 men, 22 women; age range 44–89 years, median age 71.0 years) were included (Fig. 1). Of these, 70 (86.4%) were in Child-Pugh class A; 11 (13.6%) were in class B. The final diagnoses included hypervascular HCC (n = 49), metastatic liver tumors (n = 13), HBP hypointense nodules without APHE (n = 10), and hemangiomas (n = 9). Hepatic lesions had been identified on prior imaging studies in 71 patients, whereas they were first detected on EOB-enhanced MRI in 10 patients.

### 3.2 Overall image quality

As shown in Table 1, artifact scores in the left hepatic lobe were significantly worse with ICE at both 10 and 20 min HBP (10 min: mean 3.9 vs 3.1; 20 min: 3.9 vs 3.3; all p < 0.01). Similarly, artifact scores in the right hepatic lobe were also significantly worse with ICE at both time points (10 min: mean 4.1 vs 3.6; 20 min: 4.1 vs 3.7; all p < 0.01). Interobserver agreement was good for artifact scores in both the left hepatic lobe (κrange, 0.81–0.90) and the right hepatic lobe (κrange, 0.77–0.85).

**Table 1:** Subjective image quality scores for artifacts.

	Left lobe		Right lobe	
**10 min**	ICE (-)	ICE (+)	ICE (-)	ICE (+)
Score 1	0	0	0	0
Score 2	1	8	0	3
Score 3	10	57	0	27
Score 4	64	14	71	43
Score 5	4	0	6	4
**20 min**				
Score 1	0	0	0	0
Score 2	0	2	0	1
Score 3	12	53	0	27
Score 4	64	22	71	44
Score 5	3	2	6	5

Note.—Values indicate the number of patients.

ICE: intracellular enhancement technique

**Fig. 2:**
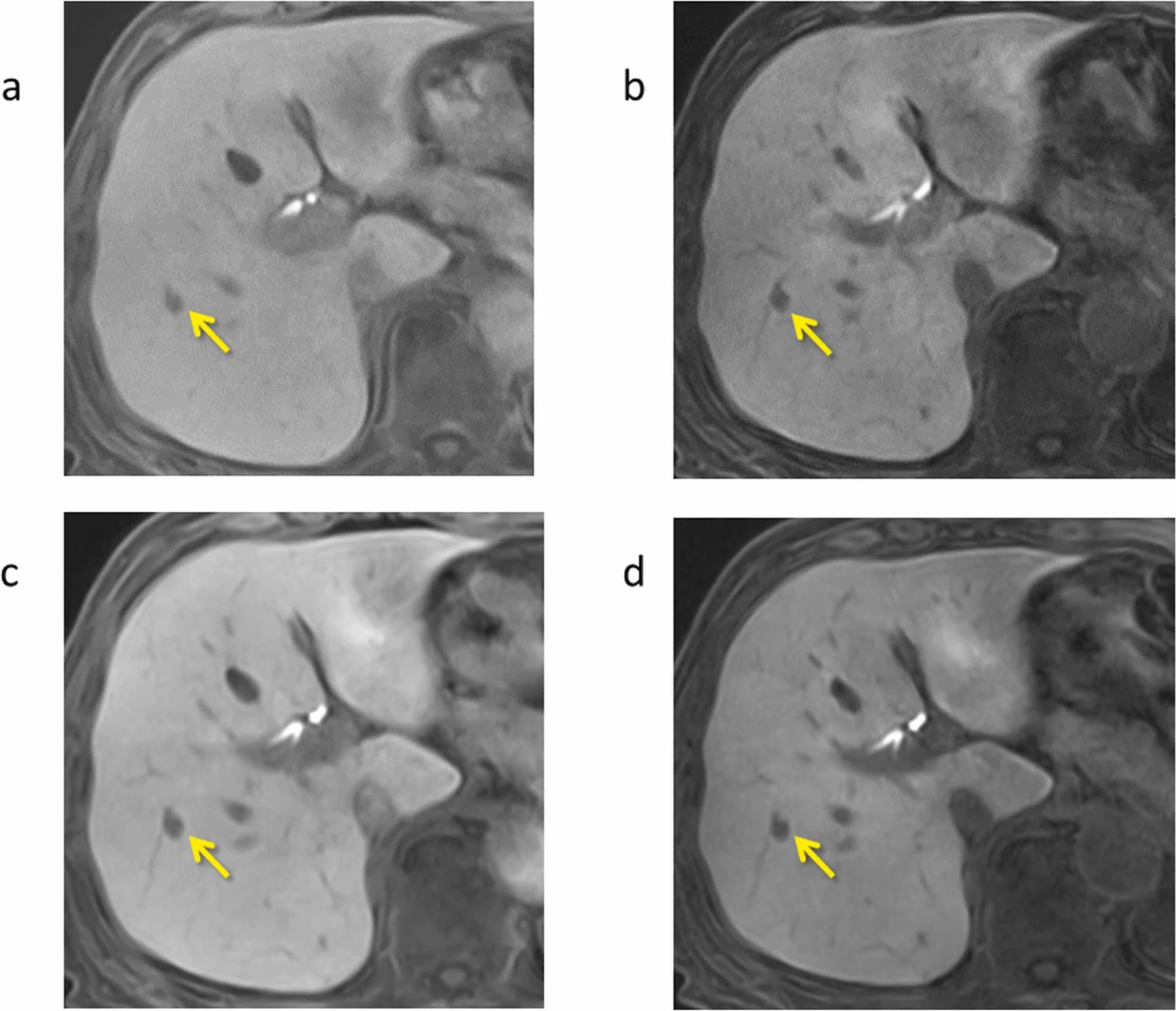
Hepatic metastasis in a 77-year-old woman with Child-Pugh class A liver function. Hepatobiliary-phase (HBP) images obtained using (a) 10-min HBP without ICE (i ^−^ ), (b) 10-min HBP with ICE (i ^+^ ), (c) 20-min HBP without ICE (i ^−^ ), and (d) 20-min HBP with ICE (i ^+^ ). ICE images ((b) and (d)) demonstrate heterogeneous liver signal intensity caused by motion-sensitized driven equilibrium (MSDE)-related artifacts, which are more pronounced in the left hepatic lobe and result in reduced overall image quality. Nevertheless, the hepatic metastasis (arrow) appears more conspicuous on the ICE images than on the corresponding conventional i^-^−20 min HBP images.

As shown in Table 2, overall image quality scores were significantly lower with ICE at both 10 and 20 min HBP (10 min: mean 3.8 vs 3.1; 20 min: 4.0 vs 3.3; all p < 0.01) (Fig. 2). Interobserver agreement for overall image quality scores was good (κrange, 0.88–0.98).

**Table 2:** Overall image quality scores.

	10 min		20 min	
	ICE (-)	ICE (+)	ICE (-)	ICE (+)
Score 1	0	0	0	0
Score 2	2	8	0	4
Score 3	17	56	7	52
Score 4	57	16	67	22
Score 5	5	1	7	3

Note.—Values indicate the number of patients.

ICE: intracellular enhancement technique

### 3.3 Lesion analysis

#### 3.3.1 Effect of ICE on lesion visibility

**Fig. 3:**
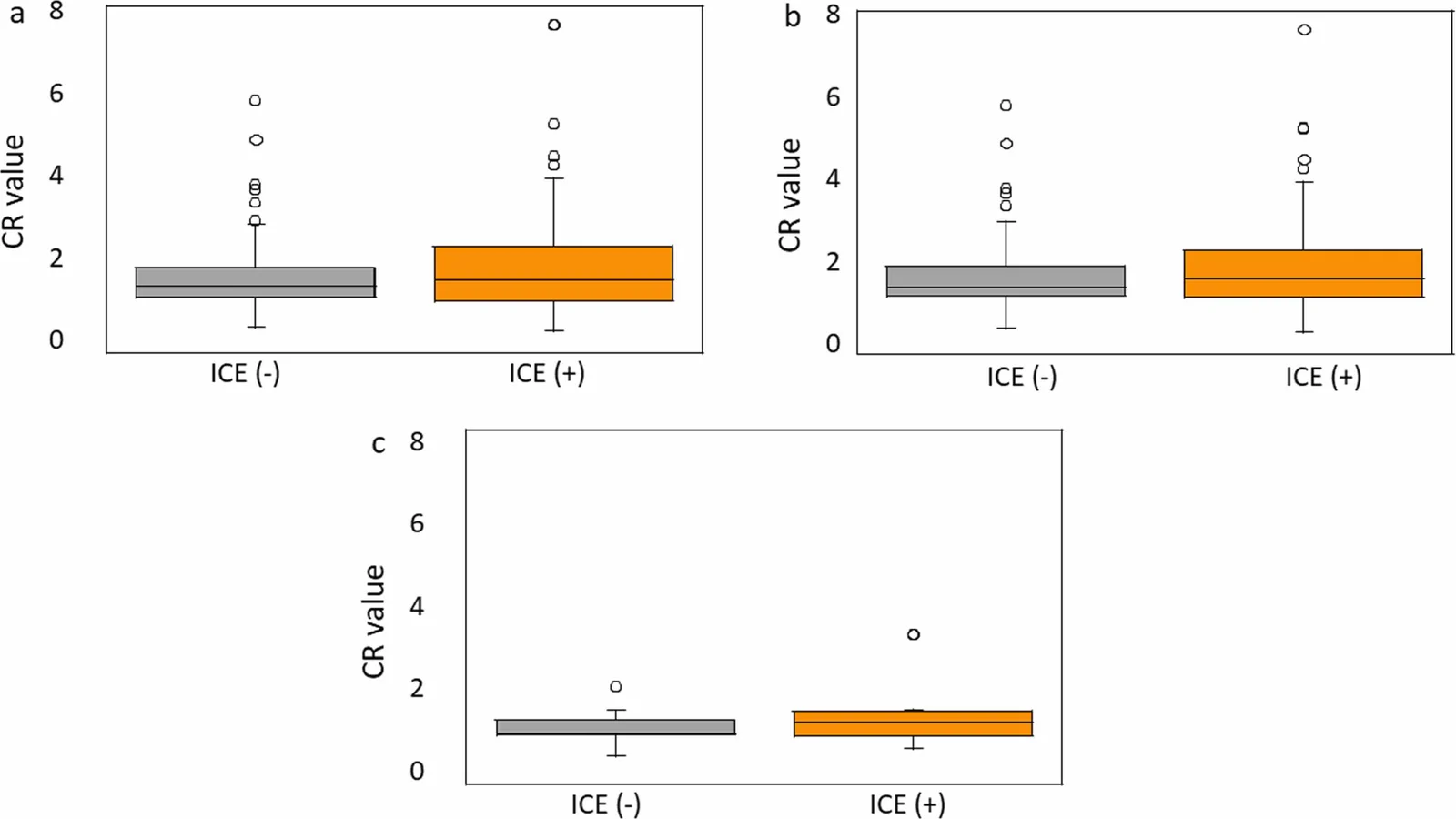
Contrast ratios on i ^+^ −20 min and conventional i^-^−20 min hepatobiliary-phase (HBP) images. Box-and-whisker plots show the contrast ratio (CR) for (a) all patients, (b) patients with Child–Pugh class A, and (c) patients with Child–Pugh class B. Boxes indicate the interquartile range, the horizontal line within each box represents the median, whiskers indicate the range, and circles represent outliers.

**Fig. 4:**
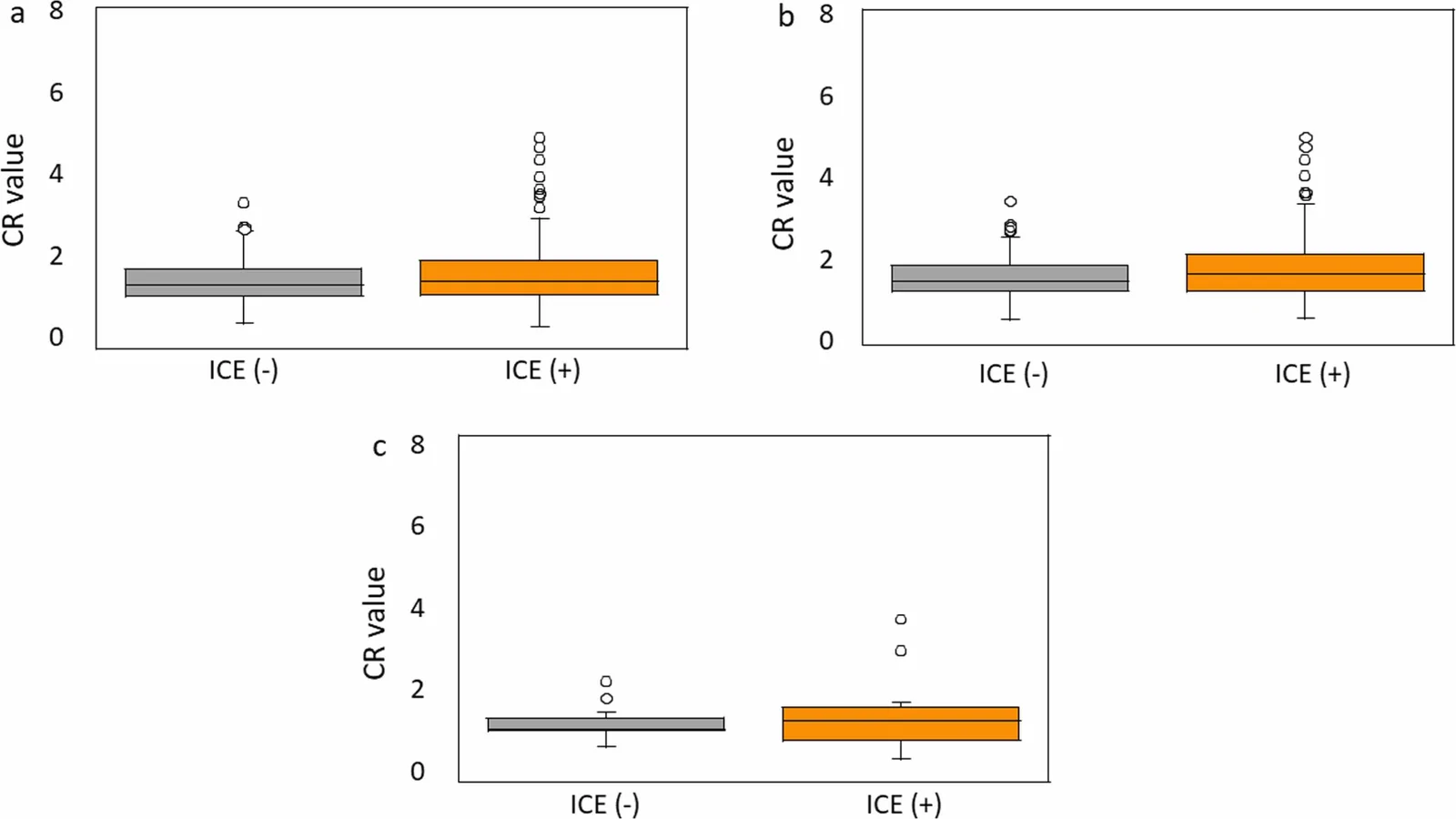
Contrast ratios on i ^+^ −10 min and conventional i^-^−10 min hepatobiliary-phase (HBP).images. Box-and-whisker plots show the contrast ratio (CR) for (a) all patients, (b) patients with Child–Pugh class A, and (c) patients with Child–Pugh class B.

**Fig. 5:**
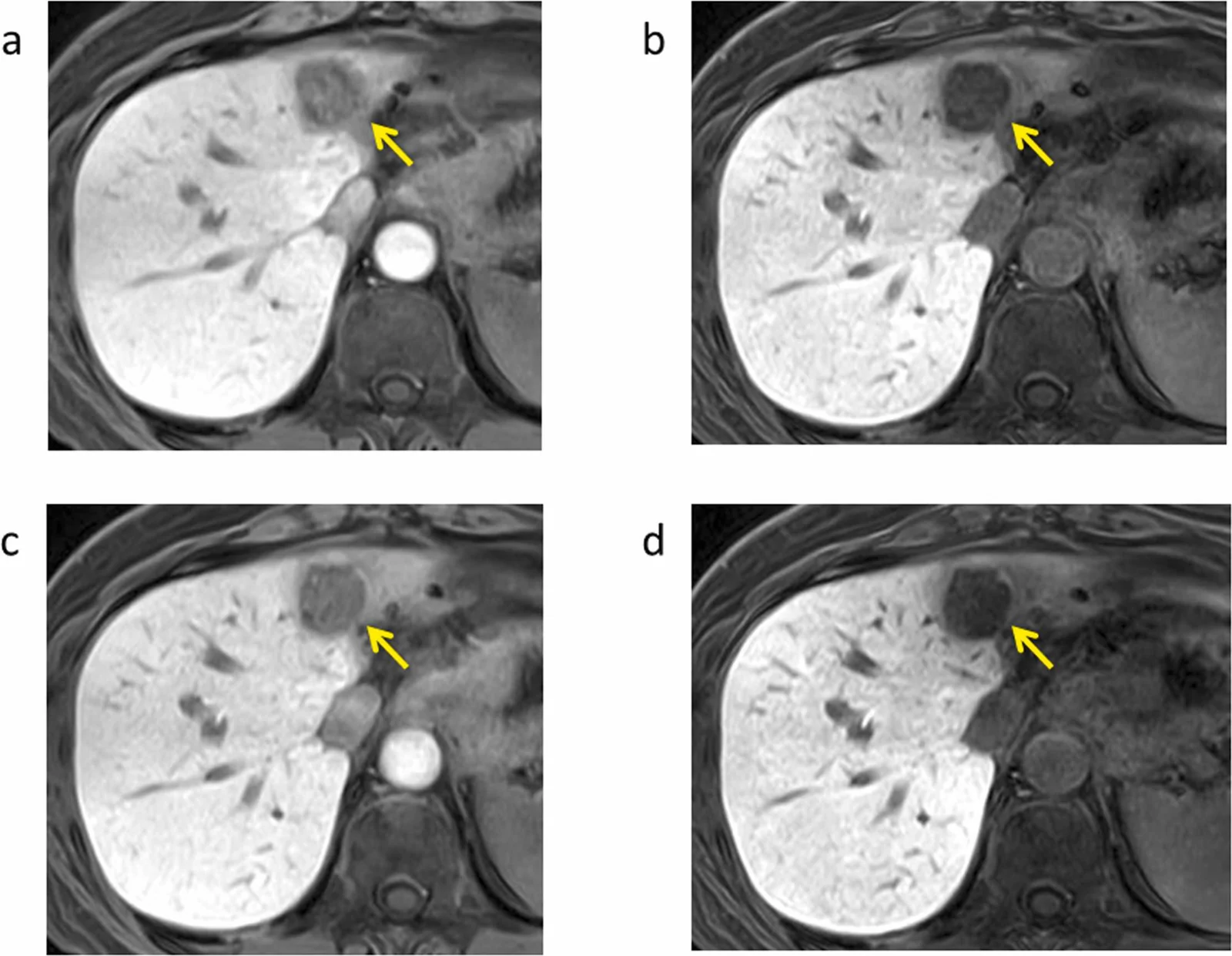
Hepatocellular carcinoma in a 60-year-old woman with Child–Pugh class A liver function. Hepatobiliary-phase images obtained using (a) 10-min HBP without ICE (i ^−^ ), (b) 10-min HBP with ICE (i ^+^ ), (c) 20-min HBP without ICE (i ^−^ ), and (d) 20-min HBP with ICE (i ^+^ ). ICE improves the contrast between the hepatocellular carcinoma (arrow) and the surrounding hepatic parenchyma on both the 10- and 20-min HBP images. In addition,the i ^+^ −10 min HBP image (b) demonstrates greater lesion conspicuity than the conventional i^-^−20 min HBP image (c). ICE images ((b) and (d)) also demonstrate heterogeneous liver signal intensity caused by motion-sensitized driven equilibrium (MSDE)-related artifacts, particularly in the left hepatic lobe, resulting in reduced overall image quality.

The median lesion size was 47.4 mm^2^ (range, 4.0–168.6 mm^2^). In all 81 patients, the CR was significantly higher with ICE at both 10 and 20 min HBP (10 min: median 1.48 vs 1.58; 20 min: median 1.54 vs 1.67; p = 0.02 and p = 0.01, respectively) (Table 3, Fig. 3, Fig. 4). Subjective lesion visibility scores were also significantly higher with ICE at both time points (10 min: mean 3.6 vs 4.1; 20 min: mean 3.9 vs 4.4; both p < 0.01) (Table 4, Fig. 2, Fig. 5).

**Table 3:** Contrast ratio on HBP scans with/without ICE.

		ICE (-)	ICE (+)	*p* value (95% CI)
10 min	All (n = 81)	1.48 (0.61–3.48)	1.58 (0.54–5.00)	0.02 (0.03–0.41)
	Child-Pugh class A (n = 70)	1.50 (0.60–3.48)	1.68 (0.62–5.01)	0.03 (0.03–0.42)
	Child-Pugh class B (n = 11)	1.20 (0.79–2.35)	1.43 (0.54–3.81)	0.55 (−0.55–0.97)
20 min	All (n = 81)	1.54 (0.61–5.87)	1.67 (0.50–7.63)	0.01 (0.06–0.50)
	Child-Pugh class A (n = 70)	1.57 (0.61–5.87)	1.76 (0.50–7.63)	0.04 (0.02–0.51)
	Child-Pugh class B (n = 11)	1.17 (0.65–2.24)	1.40 (0.81–3.46)	0.15 (−0.16–0.90)

Data are the median (range).

HBP: hepatobiliary-phase; ICE: intracellular enhancement technique; 95% CI: 95% confidence interval

**Table 4:** Subjective image quality scores for hepatic lesions for all patients.

	10 min		20 min	
	ICE (-)	ICE (+)	ICE (-)	ICE (+)
Score 1	1	1	1	1
Score 2	5	1	0	0
Score 3	28	15	15	8
Score 4	41	36	54	31
Score 5	6	28	11	41

Note.—Values indicate the number of lesions.

ICE: intracellular enhancement technique

In the subgroup analysis, patients with Child–Pugh class A showed significantly higher CR values with ICE at both 10 and 20 min HBP (10 min: median 1.50 vs 1.68; 20 min: median 1.57 vs 1.76; p = 0.03 and p = 0.04, respectively) (Table 3, Fig. 3, Fig. 4). Subjective lesion visibility scores were likewise significantly higher with ICE (10 min: mean 3.6 vs 4.2; 20 min: mean 4.0 vs 4.4; both p < 0.01) (Table 5, Fig. 2, Fig. 5).

**Table 5:** Subjective image quality scores for hepatic lesions for patients with Child-Pugh class A.

	10 min		20 min	
	ICE (-)	ICE (+)	ICE (-)	ICE (+)
Score 1	1	1	1	1
Score 2	3	1	0	0
Score 3	25	11	11	5
Score 4	35	29	47	27
Score 5	6	28	11	37

Note.—Values indicate the number of lesions.

ICE: intracellular enhancement technique

**Fig. 6:**
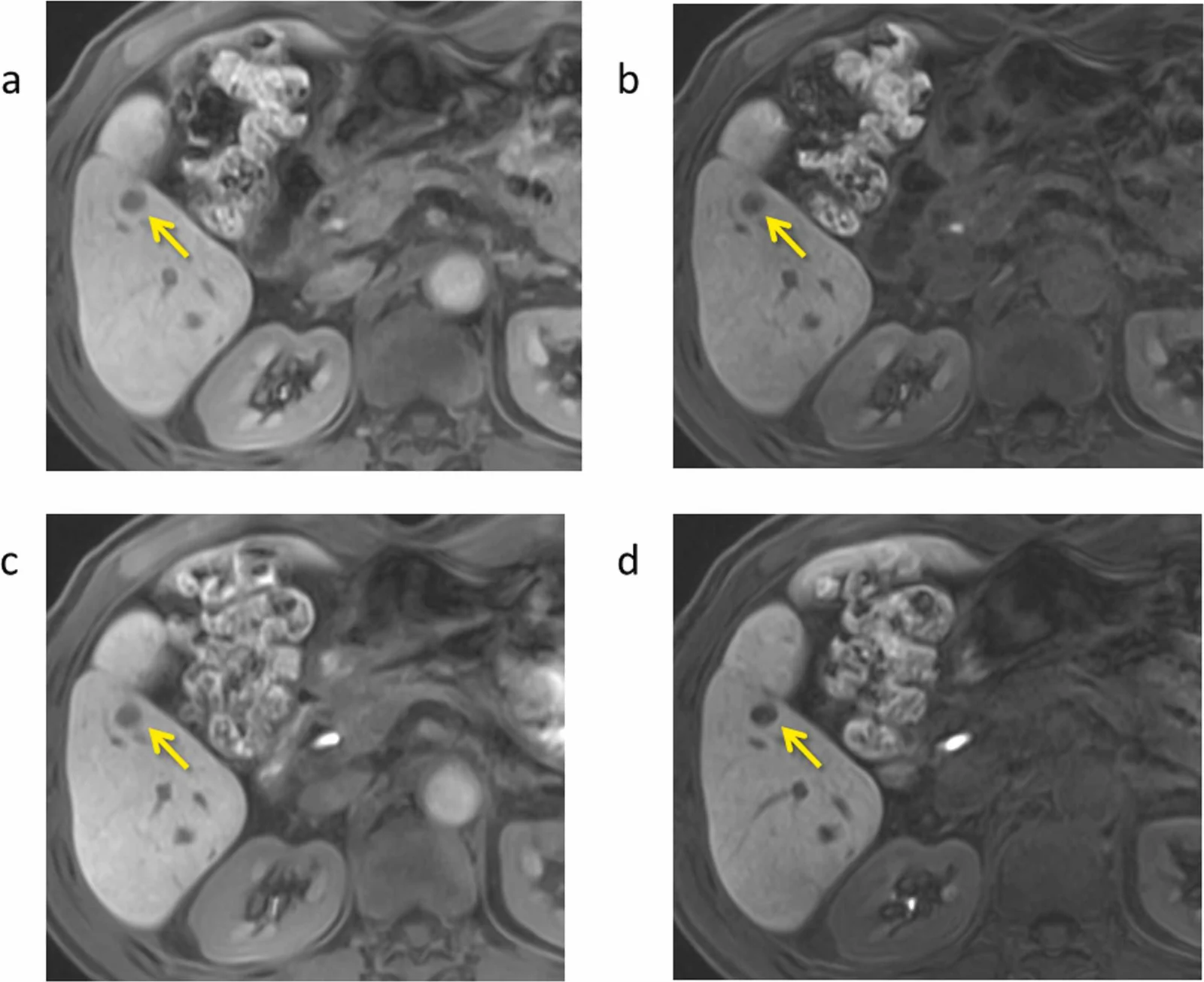
Hepatocellular carcinoma in a 62-year-old man with Child–Pugh class B liver function. Hepatobiliary-phase images obtained using (a) 10-min HBP without ICE (i ^−^ ), (b) 10-min HBP with ICE (i ^+^ ), (c) 20-min HBP without ICE (i ^−^ ), and (d) 20-min HBP with ICE (i ^+^ ). Compared with the conventional i^-^−20 min HBP image (c), lesion-to-liver contrast is slightly lower on both the i^-^−10 min HBP image (a) and the ICE i ^+^ −10 min HBP image (b), indicating that the benefit of ICE may be limited in some patients with impaired liver function.

In patients with Child–Pugh class B, CR values tended to increase with ICE; however, the differences did not reach statistical significance (10 min: median 1.20 vs 1.43, p = 0.55; 20 min: median 1.17 vs 1.40; p = 0.15) (Table 3, Fig. 3, Fig. 4). Similarly, subjective lesion visibility scores also showed no statistically significant differences (10 min: mean 3.4 vs 3.6, p = 0.08; 20 min: mean 3.6 vs 4.1, p = 0.05) (Fig. 6 and Table 6).

**Table 6:** Subjective image quality scores for hepatic lesions for patients with Child-Pugh class B.

	10 min		20 min	
	ICE (-)	ICE (+)	ICE (-)	ICE (+)
Score 1	0	0	0	0
Score 2	2	0	0	0
Score 3	3	4	4	3
Score 4	6	7	7	4
Score 5	0	0	0	4

Note.—Values indicate the number of lesions.

ICE: intracellular enhancement technique

Interobserver agreement for subjective lesion visibility scores was excellent (κrange, 0.93–0.98).

#### 3.3.2 Assessment of ICE for a reduction of delay time

**Fig. 7:**
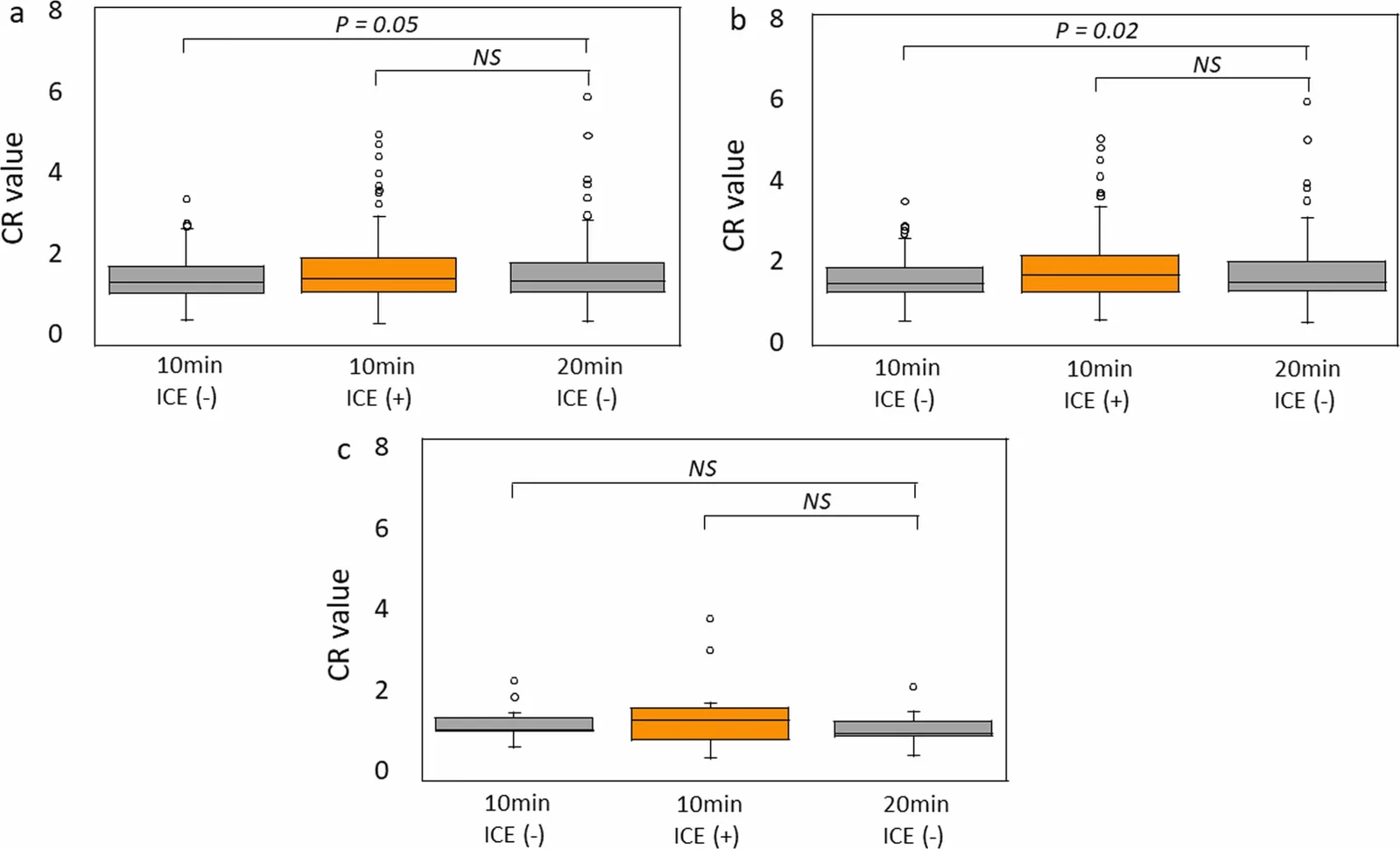
Comparison of contrast ratios among i^-^−10 min, i ^+^ −10 min, and conventional i^-^−20 min hepatobiliary-phase (HBP) images. Box-and-whisker plots show the contrast ratio (CR) for (a) all patients, (b) patients with Child–Pugh class A, and (c) patients with Child–Pugh class B. NS, not significant.

In all patients, there were no significant differences in the CR on i^-^−10 min and i^+^−10 min HBP compared to that on i^-^−20 min HBP (median 1.48 vs 1.54; p = 0.05 for i^-^−10 min HBP; median 1.58 vs 1.54; p = 0.48 for i^+^−10 min HBP) (Table 7 and Fig. 7). Visualization scores for hepatic lesions were significantly lower on i^-^−10 min HBP compared with i^-^−20 min HBP (mean 3.6 vs 3.9; p < 0.01). Moreover, visualization scores on i^+^−10 min HBP were significantly higher than those on i^-^−20 min HBP (mean 4.1 vs 3.9; p < 0.01) (Table 4).

**Table 7:** Contrast ratios: 10 min HBP with/without ICE vs. 20 min HBP without ICE.

	10 min		20 min ICE (-)	*p* value 95% CI
All (n = 81)	ICE (-)	1.48 (0.61–3.48)	1.54 (0.61–5.87)	0.05 (−0.00–0.32)
	ICE (+)	1.58 (0.54–5.00)		0.48 (−0.24–0.12)
Child-Pugh class A (n = 70)	ICE (-)	1.50 (0.60–3.48)	1.57 (0.61–5.87)	0.02 (0.02–0.37)
	ICE (+)	1.68 (0.62–5.01)		0.78 (−0.21–0.16)
Child-Pugh class B (n = 11)	ICE (-)	1.20 (0.79–2.35)	1.17 (0.65–2.24)	0.63 (−0.50–0.31)
	ICE (+)	1.43 (0.54–3.81)		0.34 (−0.98–0.37)

Data are the median (range).

HBP: hepatobiliary-phase; ICE: intracellular enhancement technique; 95% CI: 95% confidence interval

In patients with Child–Pugh class A, CR values were significantly lower on i^-^−10 min HBP than on i^-^−20 min HBP (median 1.50 vs 1.57; p = 0.02), while there was no significant difference in CR values on i^+^−10 min HBP compared to those on i^-^−20 min HBP (median 1.68 vs 1.57; p = 0.78) (Table 7 and Fig. 7). Visualization scores were significantly lower on i^-^−10 min HBP than on i^-^−20 min HBP (mean 3.6 vs 4.0; p < 0.01), whereas scores on i^+^−10 min HBP were significantly higher than those on i^-^−20 min HBP (mean 4.2 vs 4.0; p < 0.01) (Table 5, Fig. 5).

In patients with Child–Pugh class B, no significant difference was observed in CR values on both i^-^−10 min and i^+^−10 min HBP compared to those on i^-^−20 min HBP (median 1.20 vs 1.17; p = 0.63 for i^-^−10 min HBP; median 1.43 vs 1.17; p = 0.34 for i^+^−10 min HBP) (Table 7 and Fig. 7). Similarly, there were no significant differences in subjective lesion visibility scores (mean 3.4 vs 3.6; p = 0.19 for i^-^−10 min HBP; mean 3.6 vs 3.6; p = 1.00 for i^+^−10 min HBP) (Table 6 and Fig. 6).

## 4 Discussion

ICE significantly increased the CR and visualization score for hepatic lesions on both 10- and 20-min HBP images. Although no significant difference was observed in CR values on both i^-^−10 min and i^+^−10 min HBP compared to those on i^-^−20 min HBP, the visualization score for hepatic lesions was significantly lower on i^-^−10 min HBP, but higher on i^+^−10 min HBP compared to that on i^-^−20 min HBP. Consequently, by enhancing the contrast between hepatic lesions and the surrounding hepatic parenchyma, ICE can improve the visibility of hepatic lesions on the HBP and has the potential to allow for a shorter HBP delay time without compromising lesion visualization.

Increasing the flip angle (FA) improved the contrast between non-hepatocyte lesions and the hepatic parenchyma [7], [20], [21], [22]. However, a higher FA increases energy deposition expressed as the specific absorption rate (SAR). While this is usually acceptable at 1.5 T, the SAR is a significant limitation at 3 T where the baseline SAR is approximately fourfold higher [21]. The associated increase in the image noise may also degrade the diagnostic quality [23]. Our study was conducted with 3 T MRI, to which ICE can be safely applied. Although no previous studies have evaluated ICE for improving hepatic lesion conspicuity or shortening the HBP delay time, our findings suggest that ICE may provide an alternative approach to HBP optimization, particularly in the 3 T setting where increasing the FA is constrained by SAR.

Subgroup analysis revealed that ICE significantly improved the CR and visualization score for hepatic lesions at both 10- and 20-min HBP imaging in patients with Child–Pugh class A. In contrast, although CR and score tended to increase with ICE in Child–Pugh class B patients, the differences were not statistically significant. Furthermore, in patients with Child–Pugh class A, there was no significant difference in the CR between i^+^−10 min HBP and i^-^−20 min HBP, while the subjective lesion visibility score was significantly higher on i^+^−10 min HBP than on i^-^−20 min HBP. In contrast, in patients with Child–Pugh class B, neither the CR nor the subjective score showed a significant difference between i^+^−10 min HBP and i^-^−20 min HBP. Previous studies have suggested that a hepatobiliary delay time of 10 min after EOB injection is sufficient in patients with normal liver function, while the conventional 20-min delay is recommended in patients with cirrhosis [24], [25]. In this context, ICE might be expected to improve lesion visibility in patients with impaired liver function. However, in our Child–Pugh class B subgroup, the improvement was limited and did not reach statistical significance. Although the small sample size (n = 11) may have resulted in insufficient statistical power, these findings suggest that the benefit of ICE in patients with moderate liver dysfunction may be limited. Further studies with larger cohorts are needed to clarify the clinical value of ICE in patients with impaired liver function.

Because ICE incorporates an MSDE pulse, potential image degradation is a concern. We applied g-denoising, the only noise reduction algorithm available on our scanner, to both i^−^ and i^+^ HBP scans. Although g-denoising adaptively reduced the noise related to parallel imaging [16], it did not fully eliminate artifacts induced by the MSDE pulse. In fact, our results showed that artifact scores worsened and overall image quality scores decreased by ICE. Although ICE improved lesion visibility, this benefit should be interpreted in the context of the accompanying reduction in overall image quality and increase in artifacts, particularly in the left hepatic lobe. Therefore, ICE should not be regarded as a replacement for conventional HBP imaging but rather as a complementary technique that may improve lesion conspicuity in selected clinical situations. In general, contrast-to-noise ratio is conventionally calculated to quantitatively assess lesion visibility; however, it was not calculated in the present study because of heterogeneous artifacts distribution within the liver precluded accurate noise measurement. To optimize ICE for clinical use, the development of advanced artifact-reduction strategies specifically targeting MSDE-related distortions is important.

With an MSDE pulse (b = 60 s/mm²), ICE suppresses capillary vascular signals and may slightly attenuate signals due to diffusion [9], [26]. Although suppression of the extracellular space has not been definitively demonstrated [9], our results, showing that ICE improves the visibility of hepatic lesions on HBP, indicate that ICE is clinically useful.

A non-inferiority trial would be the appropriate design for comparing CR and subjective scores for hepatic tumors on i^+^−10 min HBP and i^-^−20 min HBP. Based on the current data, assuming a non-inferiority margin of 0.18 (10% of the mean CR on i^-^−20 min HBP), a one-sided alpha level of 0.05, and a statistical power of 0.80, a total sample size of 174 patients would be required. Because the present study included substantially fewer patients, a formal non-inferiority analysis was not performed. Although no significant differences were observed between i^+^−10 min HBP and i^-^−20 min HBP in all patients and patients with Child-Pugh class A, the absence of a statistically significant difference does not establish non-inferiority. Accordingly, the present findings should not be interpreted as demonstrating that i^+^−10 min HBP imaging can replace the conventional i^-^−20 min HBP protocol. Whether i^+^−10 min HBP is non-inferior to i^-^−20 min HBP should be evaluated in adequately powered future studies designed to evaluate diagnostic performance and formally test non-inferiority.

This study has several limitations. First, the study population was relatively small, and the investigation was conducted at a single institution, which may limit the generalizability of the findings. Second, because longer delay times may improve visualization of the biliary system and hepatic parenchymal enhancement in patients with advanced cirrhosis [25], the effect of ICE should also be evaluated on HBP images acquired with delays longer than 20 min in this population. Third, the use of ICE significantly increased artifacts in both hepatic lobes, with the effect appearing more pronounced in the left lobe (Fig. 2, Fig. 5). Although this suggests a potential adverse impact on visibility of left-lobe lesions, lesion visibility was actually improved with ICE even for lesions located in the left lobe (Fig. 5). However, because the number of left-lobe lesions in this study was small, lesion visibility and quantitative parameters were not analyzed separately by lesion location. In addition, quantitative assessment of left hepatic lobe artifacts was not feasible because the artifacts were irregularly distributed and did not permit standardized ROI placement. Therefore, the influence of lesion location should be investigated in future studies with larger sample sizes. Fourth, although this study demonstrated improved lesion visibility with ICE, we did not perform reader-based diagnostic performance analyses, such as receiver operating characteristic analysis, nor was the study designed or powered to formally evaluate non-inferiority of the i^+^−10 min protocol compared with the conventional i^-^−20 min protocol. Therefore, future adequately powered studies evaluating diagnostic performance and formal non-inferiority are warranted. Fifth, we did not categorize the lesions by type; however, only lesions demonstrating hypointensity on HBP were included, suggesting that our results are at least applicable to HBP-hypointense lesions. Finally, liver function was classified using the Child–Pugh system, which is widely used; however, this classification cannot distinguish between normal liver function and mild liver dysfunction, and both are therefore included in Child–Pugh class A. As a result, the effects observed in Child–Pugh class A in the present study may have been influenced by patients with normal liver function, and it remains unclear whether similar effects can be expected in patients with mild liver dysfunction.

In conclusion, although the ICE technique increases image artifacts and modestly reduces overall image quality, it can improve the visibility of hepatic lesions on HBP, particularly in patients with preserved liver function. Moreover, it has the potential to reduce delay time without compromising lesion visualization; however, further studies with larger sample sizes, including non-inferiority trials, are required to validate the feasibility of delay time reduction.

## CRediT authorship contribution statement

**Masataka Tsuge:** Resources. **Toru Higaki:** Software, Resources, Formal analysis. **Yuko Nakamura:** Writing – review & editing, Writing – original draft, Supervision, Methodology, Funding acquisition, Formal analysis, Data curation, Conceptualization. **Kazuo Awai:** Supervision, Project administration, Funding acquisition, Conceptualization. **Masahiro Takizawa:** Software. **Shiro Oka:** Resources. **Takashi Nishihara:** Software. **Shogo Maeda:** Writing – original draft, Formal analysis, Data curation. **Yukiko Honda:** Resources, Data curation. **Shota Kondo:** Resources, Data curation. **Tomokazu Kawaoka:** Resources. **Yuji Akiyama:** Resources. **Hirokazu Asaka:** Software. **Yoshitaka Bito:** Software, Methodology. **Dara Fonseca:** Resources, Data curation. **Kenji Kodakari:** Resources, Data curation.

## Informed consent and patient details

The authors declare that this report does not contain any personal information that could lead to the identification of the patients.

## Ethics statement

Each subject signed an informed consent form approved by Ethical Committee for Epidemiology of Hiroshima University (Hiroshima, Japan), and the study was performed in accordance with the Declaration of Helsinki of the World Medical Association.

## Declaration of Generative AI use

Generative AI was used only for English language editing (grammar, wording, and clarity). No generative AI was used for data collection, figure/table generation, or drawing scientific conclusions. The authors reviewed and take full responsibility for all content.

## Funding

YN received a research grant from 10.13039/501100002424FUJIFILM Corporation (grant number:0G20KA7113). FUJIFILM Corporation provided technical support but had no role in the study design, data acquisition, image interpretation, statistical analysis, manuscript preparation, or the decision to submit the manuscript for publication. The authors retained full control over the study design, data analysis, interpretation of the results, and the decision to publish.

## Declaration of Competing Interest

The authors declare the following financial interests/personal relationships which may be considered as potential competing interests: Yuko Nakamura reports financial support was provided by FUJIFILM Corporation. Masahiro Takizawa reports financial support was provided by FUJIFILM Corporation. Hirokazu Asaka reports financial support was provided by FUJIFILM Corporation. Takashi Nishihara reports financial support was provided by FUJIFILM Corporation. Yoshitaka Bito reports financial support was provided by FUJIFILM Corporation. If there are other authors, they declare that they have no known competing financial interests or personal relationships that could have appeared to influence the work reported in this paper.

## Data Availability

The data that support the findings of this study are not openly available due to reasons of sensitivity and are available from the corresponding author upon reasonable request.
